# Large linear magnetoresistance in heavily-doped Nb:SrTiO_3_ epitaxial thin films

**DOI:** 10.1038/srep34295

**Published:** 2016-10-05

**Authors:** Hyunwoo Jin, Keundong Lee, Seung-Hyub Baek, Jin-Sang Kim, Byung-ki Cheong, Bae Ho Park, Sungwon Yoon, B. J. Suh, Changyoung Kim, S. S. A. Seo, Suyoun Lee

**Affiliations:** 1 Center for Electronic Materials, Korea Institute of Science and Technology, Seoul 136-791, Korea; 2 Institute of Physics and Applied Physics, Yonsei University, Seoul 120-749, Korea; 3 Department of Physics and Astronomy, Institute of Applied Physics, Research Institute of Advanced Materials (RIAM), Seoul National University, Seoul 151-747, Korea; 4 Department of Nanomaterials Science and Technology, University of Science and Technology, Daejeon, 305-333, Republic of Korea; 5 Department of Physics, Konkuk University, Seoul 143-701, Korea; 6 Department of Physics, The Catholic University of Korea, Bucheon 420-743, Korea; 7 Department of Physics and Astronomy, University of Kentucky, Lexington, KY 40506, USA

## Abstract

Interaction between electrons has long been a focused topic in condensed-matter physics since it has led to the discoveries of astonishing phenomena, for example, high-*T*_c_ superconductivity and colossal magnetoresistance (CMR) in strongly-correlated materials. In the study of strongly-correlated perovskite oxides, Nb-doped SrTiO_3_ (Nb:SrTiO_3_) has been a workhorse not only as a conducting substrate, but also as a host possessing high carrier mobility. In this work, we report the observations of large linear magnetoresistance (LMR) and the metal-to-insulator transition (MIT) induced by magnetic field in heavily-doped Nb:STO (SrNb_0.2_Ti_0.8_O_3_) epitaxial thin films. These phenomena are associated with the interplay between the large classical MR due to high carrier mobility and the electronic localization effect due to strong spin-orbit coupling, implying that heavily Nb-doped Sr(Nb_0.2_Ti_0.8_)O_3_ is promising for the application in spintronic devices.

Recent discoveries of a high mobility (*μ*) two-dimensional electron gas (2DEG), superconductivity, and ferromagnetism at the interface of SrTiO_3_ (STO) with other oxides such as LaAlO_3_ and LaTiO_3_^1^,^2^,^3^,^4^, have attracted interest in the properties of STO and doped STO. Conducting Nb-doped STO has also been studied as a potential candidate for creating a high-*μ* 2DEG by using a *δ*-doped quantum well structure^5^. Previous studies have been conducted in a limited range of Nb concentration (0 ~ 5 at.%)^6^,^7^,^8^,^9^,^10^,^11^, where *μ* was observed to be ~22,000 cm^2^/Vs at 4 K for Nb concentrations of 0.02 at. % and decrease with increasing Nb concentration. In the heavy doping regime above 5 at. %, however, *μ* barely changes as a function of the Nb concentration contrary to the result of the low Nb concentration^11^. On the other hand, heavily Nb-doped STO (20 at. % Nb) has been reported to show intriguing properties such as large thermoelectric power^12^. Therefore, a deeper exploration into the effect of high-concentration doping on the carrier transport is needed to understand the physical properties of Nb-doped STO. In this work, we have investigated the magnetotransport properties of a heavily Nb-doped (~20 at.%) STO (Nb:STO) epitaxial thin film and observed intriguing phenomena, large linear magnetoresistance (LMR) and metal-to-insulator transition (MIT) induced by the magnetic field, which are unprecedented in Nb:STO with low Nb concentration. We also present a few evidences supporting that those phenomena are associated with the interplay between the large classical MR due to high carrier mobility and the localization effect due to strong spin-orbit coupling.

Sr(Nb_0.2_Ti_0.8_)O_3_ thin films are grown on STO (001) substrates by pulsed laser deposition (PLD). The Sr(Nb_0.2_Ti_0.8_)O_3_ polycrystalline target with 20 at.% Nb is prepared by a conventional solid-state reaction technique. During the film growth, the laser power, the laser repetition rate, the substrate temperature, and the oxygen partial pressure are 1.6 J/cm^2^, 2 Hz, 700 °C, and 1 × 10^−5 ^Torr, respectively. The composition of the grown film is found to be Sr:Ti:Nb = 1:0.79:0.21 using the Rutherford back scattering (RBS) method. We have examined the homogeneity of a film using the scanning electron microscopy with energy dispersive X-ray spectroscopy (SEM/EDX, Supplemental Material S1). For the measurement of electrical properties, platinum is deposited by e-beam evaporation through a shadow mask to form the electrode pattern shown in Fig. 1(b). The longitudinal resistance (*R*_*xx*_ = *V*_23_/*I*_14_) and Hall resistance (*R*_*xy*_ = *V*_65_/*I*_14_) are measured with the excitation current of 100 μA using a Source-Measure Unit (Keithley 2612A) and a nano-voltmeter (Keithley 2182). With this setup, the contacts are confirmed to be Ohmic by measuring the two probe resistance (*V*_14_/*I*_14_). The electrical properties have been investigated as a function of temperature (*T*) (1.8 K ~ 300 K) and magnetic field (*B*) to 9 T using a commercial cryogen-free cryostat (CMag Vari-9, Cryomagnetics Inc.).

Figure 1(a) shows the X-ray diffraction *θ*–2*θ* scan of a 65 nm-thick Sr(Nb_0.2_Ti_0.8_)O_3_ film near (002) peak of SrTiO_3_, which indicates that the Sr(Nb_0.2_Ti_0.8_)O_3_ films are epitaxial with the *c*-axis along the surface normal direction without any features of secondary phases or phase segregations. The observed fringe pattern indicates the atomically smooth surface of our samples. From the fringe pattern, the thickness of the film is estimated to be about 65 nm consistent with the nominal thickness. The rocking curve of the (002) peak of a Sr(Nb_0.2_Ti_0.8_)O_3_ film with the full width at half maximum (FWHM) of 0.046° indicates good crystallinity, as shown in the inset.

Figure 1(b) shows the basic transport properties of a 65 nm-thick Sr(Nb_0.2_Ti_0.8_)O_3_ film as a function of *T*, where the carrier density (*n*) is obtained by Hall measurement with *B* of ±2 T. The data displays a few intriguing features which have not been observed in lightly doped Nb:STO. First, note that, despite a huge *n* (~10^21 ^cm^−3^), *μ* is unexpectedly high at low temperature (~14,000 cm^2^/Vs at 1.8 K) resulting in a remarkably low resistivity (*ρ*) of about 8 × 10^−8^ Ωcm at 1.8 K. It is a quite unexpected observation since *μ* was reported to be 419 cm^2^/Vs and 316 cm^2^/Vs for Sr(Nb_0.01_Ti_0.99_)O_3_ and Sr(Nb_0.02_Ti_0.98_)O_3_, respectively^11^. Therefore, the observed high *μ* implies that there is a change in the transport mechanism or in the electronic band structure of the heavily-doped Nb:STO films. Below 100 K, the dependence of *μ* on *T* fits quite well to the Fermi liquid theory, 1/*μ*(*T*) = *α* + *βT*^2^ln(1/*T*) where *α* and *β* are constants^13^ meaning that Sr(Nb_0.2_Ti_0.8_)O_3_ films are degenerate. The deviation from the fit above 100 K could originate from the contribution of phonon scattering at high temperature^14^. Or, it might be associated with the phase transition of STO from the tetragonal to the cubic phase above 105 K^15^,^16^. This fact might lead to the emergence of multiple electronic bands. Indeed, as will be discussed in the later part, a fingerprint of the existence of multiple types of carriers is shown in the *B*-dependence of the Hall resistance (*R*_*xy*_), unveiling one type of carrier having remarkably high *μ* at low temperature. Therefore, we believe that such a high *μ* originates from the change in the electronic band structure without ruling out other possibilities, for example, the effect of strain.

Another intriguing feature is shown in the non-monotonic *T*-dependence of *n*. While *n* decreases with lowering *T* down to 100 K due to the reduction of thermal energy, it shows the opposite behavior below 100 K. This increase in *n* is associated with the increased dielectric screening due to the drastic increase of the dielectric permittivity of STO. In fact, a similar increase of *n* has been reported in oxygen vacancy (VO)-doped STO^17^.

In Fig. 2(a), the magnetoresistance (MR = *R*(*B*)/*R*(0)) a 65 nm-thick is plotted as a function of *B* at 1.8 K for two different orientations of *B*. Note that both MR vs. *B* curves are quite linear irrespective of the orientation although the amplitude of MR is about three-times higher for the case of the perpendicular *B*. In this work, we have investigated three samples with varying thickness (65 ~ 88 nm) and all investigated samples are shown to reproduce the LMR (Supplemental Material S2). As *T* increases, the linear dependence of MR turns into the classical quadratic dependence as shown in Fig. 2(b).

Since the first report of LMR in Bi crystals^18^, LMR have attracted much interest because MR is expected to be an even function of *B* owing to symmetry. Nevertheless, LMR has been reported for various materials leading to the development of several theoretical models (for a review, see refs 19,20 and references therein). Recently, the interest has been revived due to the observation of LMR in topological insulators^21^,^22^, multilayer epitaxial graphene^23^, and Dirac semimetals such as Cd_3_As_2_^24^,^25^. I. M. Lifshitz and V. G. Peschanskii explained the LMR using the classical electron trajectories in *B* when the material has an open Fermi surface^26^. For materials with a closed Fermi surface, A. A. Abrikosov has developed a quantum mechanical picture for the LMR^27^. According to his picture, in the quantum limit where the Landau level spacing is much larger than the thermal energy (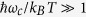, where 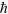, *ω*_c_, and *k*_B_ are reduced Planck constant (*h*/2π), cyclotron frequency, and Boltzmann constant, respectively), only the lowest Landau level is occupied by electrons leading to the LMR. This LMR is dubbed as “quantum LMR (QLMR)”. About 30 years later, the LMR was observed in Ag_2+δ_Te and Ag_2+δ_Se under a low *B* down to 10 Oe and high *T* up to 300 K, which does not satisfy the quantum limit criterion^28^. Again, based on his QLMR picture, A. A. Abrikosov also showed that the LMR could be observed at high *T* with low *B* under assumptions of (1) a gapless semiconductor with a linear energy vs. momentum (*E* vs. *k*) relation and (2) inhomogeneous carrier distribution^19^,^29^. As an alternative explanation, M. M. Parish and P. B. Littlewood also showed that the LMR could appear in inhomogeneous materials making the Hall effect involved in the calculation of the longitudinal resistance^30^,^31^.

In order to clarify the origin of the LMR in our samples, *T*-dependence of *ρ* and *R*_xy_ are investigated with varying *B*. Figure 3(a) shows *ρ* vs. *T* curves at various *B*. Note that, under a sufficiently strong *B*, *ρ*(*T*) shows a metal-insulator transition (MIT) at a certain temperature which increases with *B* (Fig. 3(b)). The insulating nature under a strong *B* at low *T* is also verified by the non-linear current (*I*)-voltage (*V*) characteristics at 2 K (Fig. 3(c)) in comparison with the linear *I*-*V* curves at 10 K with varying *B* (Fig. 3(d)). In addition, we have found that, below the MIT temperature, the temperature dependence of the resistivity can be described by 
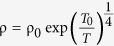
 as shown in Fig. 3(e), implying that the carrier transport is dominated by the variable-range hopping (VRH)^32^. It means that the carriers become localized by the magnetic field, invoking the effect of the weak antilocalization (WAL)^33^,^34^,^35^,^36^.

In the three dimensional WAL, MR 
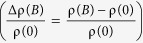
 can be described by Fukuyama-Hoshino (FH) model^37^,^38^,^39^ which is expressed as the following equation (Eq. (1)).


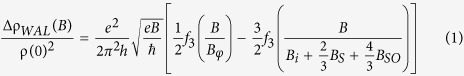


where


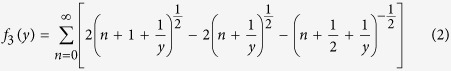


In Eq. (1), *B*_*i*_, *B*_*S*_, and *B*_SO_ are the characteristic field for inelastic, spin-flip, and spin-orbit scattering, respectively. *B*_*ϕ*_ is the characteristic dephasing field given by *B*_*i*_ + 2*B*_*S*_. Assuming that the measured MR is given by a sum of Δ*ρ*_WAL_ and the classical MR (Δ*ρ*_orb_ ~ *B*^2^) due to Lorentz effect^38^, we have fitted the observed MR as shown in Fig. 3(f). Note that the model well describes the observed LMR resulting in an estimation of the fitting parameters, *B*_*ϕ*_  = 0.112 T and *B*_SO_ = 5.618 T. These values are consistent with the result previously obtained in the LaAlO_3_/SrTiO_3_ heterojunction in a high carrier density regime induced by the electric field effect^40^, supporting the assumed model (*Δρ* = *Δρ*_WAL_ + *Δρ*_orb._) as the origin of the observed LMR.

As another check of the localization by the magnetic field, we have investigated *R*_xy_ as a function of *B* at various temperatures (Fig. 4(a)). Note that the curve is nonlinear at high temperatures (10 K ~ 50 K) in contrast to the linear dependence at low temperatures (below 10 K). The nonlinear *R*_xy_ vs. *B* curve indicates the existence of multiple electron bands. By using the two-band model for *ρ* and *R*_xy_^41^, we calculate *n* and *μ* of the two bands, (*n*_1_, *μ*_1_) and (*n*_2_, *μ*_2_), which are plotted in Fig. 4(b,c), respectively. The curve at 2 K is nearly linear resulting in erroneous values of *n*_2_ and *μ*_2_, which are reflected by big error bars. Nevertheless, it is apparent that *n*_2_ consistently decreases resulting in ~10^3^-fold reduction with lowering *T* from 50 K to 2 K. On the other hand, *n*_1_ increases by about one order with lowering *T*, leading to an overall reduction of the carrier density (*n* = *n*_1_ + *n*_2_). Furthermore, we have investigated the *T*-dependence of *n* under high magnetic field (±5 T) as shown in Fig. 4(d). Below 20 K, a drastic reduction of *n*(*T*)_5T_ is observed in contrast to the continued increase of *n*(*T*)_2T_, which provides a clear evidence of the carrier localization induced by the magnetic field.

The resurgence of *n*(*T*)_5T_ below 10 K is also intriguing, which might imply that another mechanism or new phase sets in at low temperatures under strong *B*-field. We have also investigated the magnetic properties to find a drastic increase in the magnetization (*M*) below 10 K and a little magnetic hysteresis at 5 K (see Supplemental Material S3). It seems to imply that the observed resurgence of *n*(*T*)_5T_ below 10 K is associated with a possible emergence of an unprecedented electronic and magnetic phase in the heavily-doped Nb:STO films.

The above results, that are, (1) VRH-dominated transport under strong magnetic field, (2) the MR described by the FH model for the WAL, and (3) decrease in *n* under strong magnetic field, seem to indicate that the large LMR observed in Sr(Nb_0.2_Ti_0.8_)O_3_ results from the interplay between a large classical MR due to the high carrier mobility and the localization effect due to strong spin-orbit coupling. As other possibilities, the role of inhomogeneity suggested by M. M. Parish and P. B. Littlewood^29^,^30^ is ruled out based on the observation of homogeneous distribution of components as confirmed by the SEM/EDX (see Supplemental Material S1). On the other hand, an interpretation in terms of the QLMR model is not ruled out supposing that Sr(Nb_0.2_Ti_0.8_)O_3_ should be a gapless material with a linear energy-momentum relation and have inhomogeneous carrier distribution. According to the QLMR model, the normalized MR, 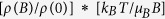, is known to approach to a constant value, which depends on the material, in the quantum limit and does not depend on *T*^33^. It has been tested and found to be true in our case up to 10 K (Figure S4 in the Supplemental Material). Therefore, we cannot rule out the QLMR although there are many evidences supporting the interplay between the classical MR and the WAL as the origin for the observed large LMR in heavily-doped Sr(Nb_0.2_Ti_0.8_)O_3_.

To summarize, we have observed the non-saturating LMR at low temperatures (below 20 K) and MIT induced by magnetic field in heavily-doped Sr(Nb_0.2_Ti_0.8_)O_3_ epitaxial thin films grown on SrTiO_3_. In addition, this material is featured by very low electrical resistivity (~8 × 10^−8^ Ωcm at 1.8 K) and high carrier mobility (~14,000 cm^2^/Vs at 1.8 K), far exceeding an expectation obtained by an extrapolation from low Nb concentration regime. We propose that the LMR is associated with the interplay between the large classical MR due to high carrier mobility and the localization effect due to strong spin-orbit coupling. Conversely, it means that the investigated Sr(Nb_0.2_Ti_0.8_)O_3_ thin film possesses the high carrier mobility and the strong spin-orbit coupling simultaneously, which imply a long spin diffusion length and an ability to effectively modulate electron’s spin, respectively. Therefore, we believe that Sr(Nb_0.2_Ti_0.8_)O_3_ is a promising channel material for the application in spintronic devices although further exploration is needed into the heavily doped Nb:STO. A study on the dependence on the Nb concentration is ongoing, which will be presented in near future.

## Additional Information

**How to cite this article**: Jin, H. *et al*. Large linear magnetoresistance in heavily-doped Nb:SrTiO_3_ epitaxial thin films. *Sci. Rep.* **6**, 34295; doi: 10.1038/srep34295 (2016).

## Supplementary Material

Supplementary Information

## Figures and Tables

**Figure 1:**
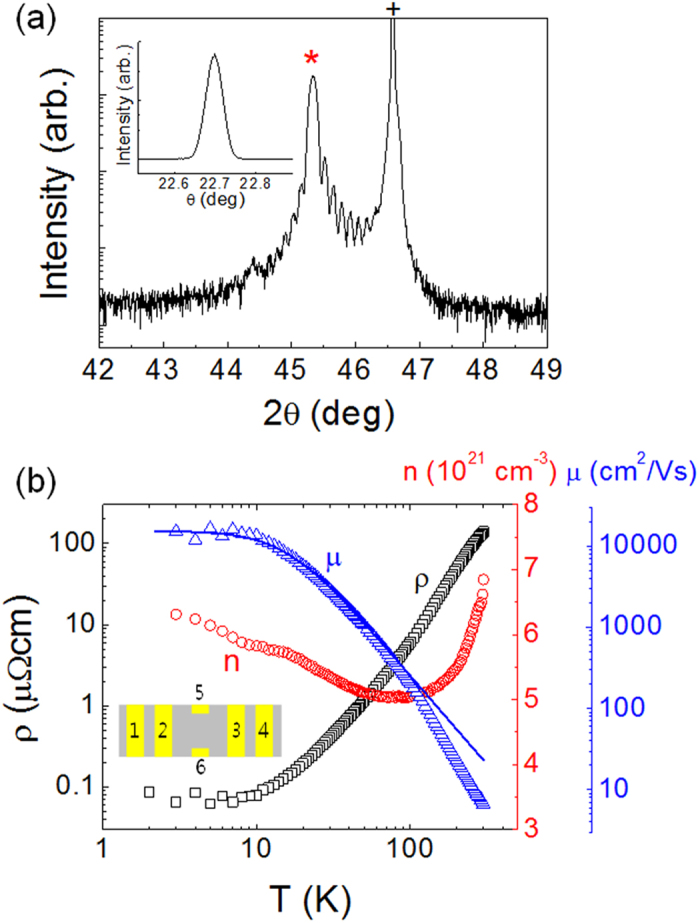
(**a**) X-ray diffraction *θ*–2*θ* scan of a 65 nm thick Sr(Nb_0.2_Ti_0.8_)O_3_ film grown on a STO (100) substrate by PLD. The black cross and red asterisk represent STO and Sr(Nb_0.2_Ti_0.8_)O_3_, respectively. The inset shows the X-ray rocking curve. (**b**) Resistivity (*ρ*, black square), carrier density (*n*, red circle), and the carrier mobility (*μ*, blue triangle) as a function of *T*. *n* was obtained by Hall measurement under ±2 T. The blue solid line is a fitting curve to the Fermi liquid theory, 1/*μ*(*T*) = *α* + *βT*^2^ln(1/*T*), where *α* and *β* are constants. The inset shows a schematic illustration of the film (grey) and the electrodes (yellow) for the electrical characterization.

**Figure 2:**
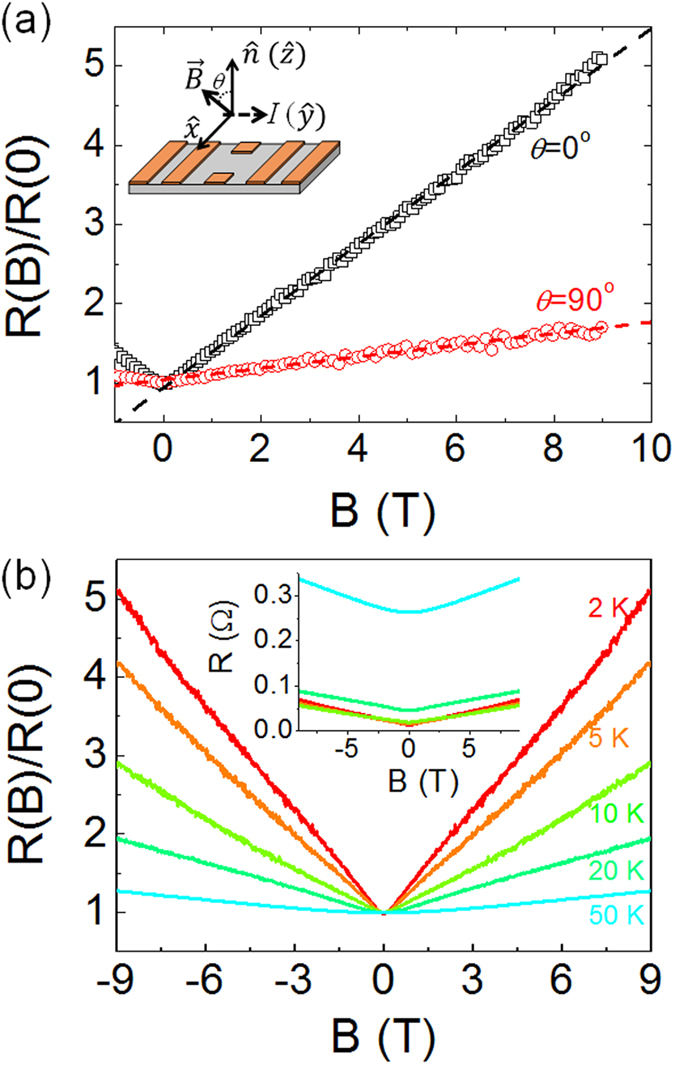
(**a**) MR a 65 nm-thick Sr(Nb_0.2_Ti_0.8_)O_3_ film as a function of *B* for *B*//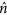 (black square) and *B*⊥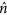 (red circle). Dashed lines represent the linear fitting curves to each data. Inset shows the definition of *θ*. (**b**) Temperature dependence of MR for *B*//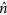. Inset shows the resistance as a function of *B* at various temperatures.

**Figure 3:**
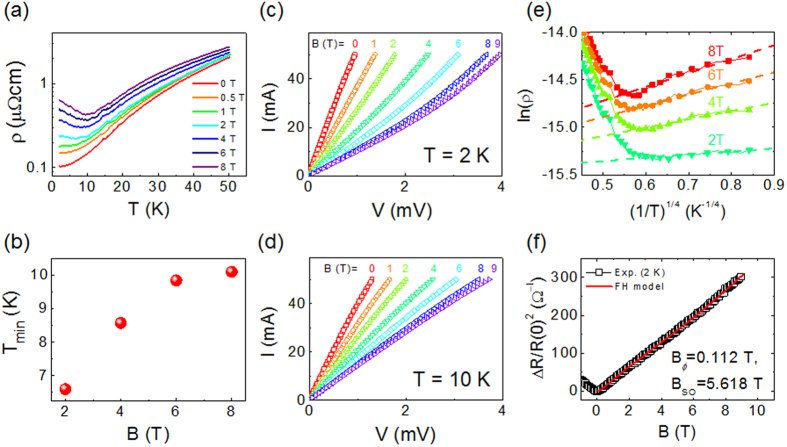
(**a**) Resistivity (*ρ*) of a 65 nm-thick Sr(Nb_0.2_Ti_0.8_)O_3_ film as a function of temperature (*T*) with varying magnetic field (*B*), (**b**) Temperature at the resistance minimum (*T*_min_) as a function of *B*, (**c**,**d**) the current (*I*)-voltage (*V*) characteristic curve with varying *B* at 2 K and 10 K, respectively. (**e**) ln(*ρ*) vs. (1/*T*)^1/4^ curves under various magnetic field. Solid lines are linear fitting to each curve below the *T*_min_. (**f**) *ΔR*/*R*(0)^2^ as a function of *B* at 2 K (replotted from Fig. 2(b)). A red solid line is the fitting curve by Fukuyama-Hoshino model (see the text) with the fitting parameters, *B*_*ϕ*_ = 0.112 T and *B*_SO_ = 5.618 T.

**Figure 4:**
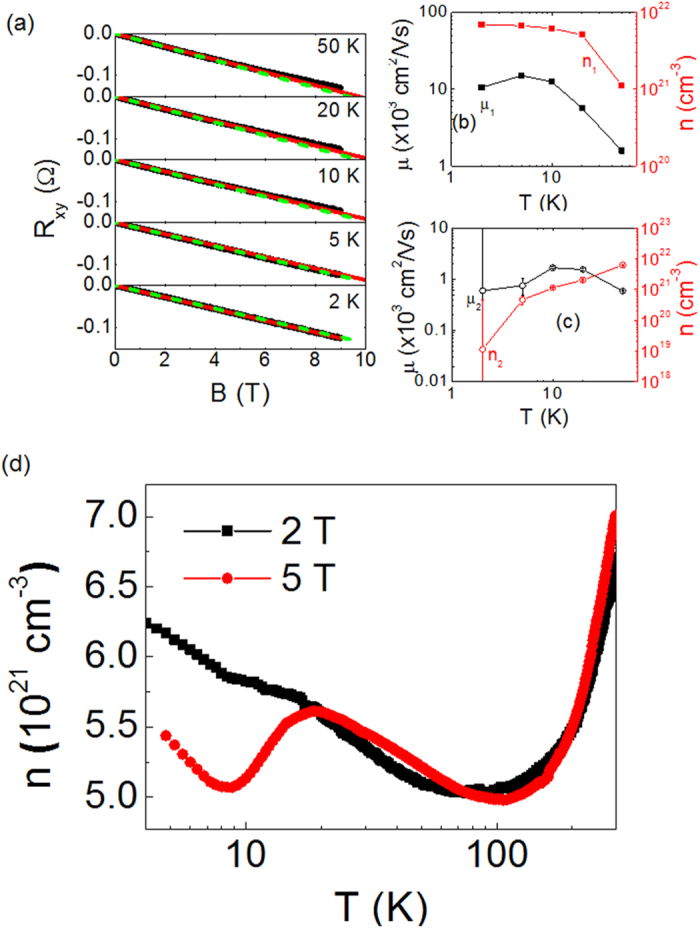
(**a**) Temperature dependence of *R*_xy_ vs. *B* curve for *B*//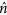. Black, red solid lines, and green dashed line represent the experimental data, the fitting curve by two-band model, and the linear fitting curve in the range of [0, 2 T], respectively. (**b**,**c**) Temperature dependence of carrier density (*n*) and carrier mobility (*μ*) of the two bands calculated by fitting to two-band model. Error bars in (**c**) represent the standard deviation while they are smaller than the size of the symbol in (**b**). (**d**) Temperature dependence of *n* measured with *B* = ±2 T (black square) and ±5 T (red circle).
