# Supplementary material for: Large linear magnetoresistance in heavily-doped Nb:SrTiO3 epitaxial thin films
Source: Sci Rep. 2016 Oct 5;6:34295. doi: 10.1038/srep34295 (PMC5050504; doi:10.1038/srep34295)
Supplement: Supplementary Information [file srep34295-s1.pdf]

Supplemental Material for

Large linear magnetoresistance in heavily-doped Nb:$SrTiO_3$ epitaxial thin films

Hyunwoo Jin[1,2], Keundong Lee[3], Seung-Hyub Baek[1,4], Jin-Sang Kim[1], Byung-ki Cheong[1], Bae Ho Park[5], Sungwon Yoon[6], B. J. Suh[6], Changyoung Kim[2], S. S. A. Seo[7], and Suyoun Lee[1,4]

[1] *Center for Electronic Materials, Korea Institute of Science and Technology, Seoul 136-791, Korea*

[2] *Institute of Physics and Applied Physics, Yonsei University, Seoul 120-749, Korea*

[3] *Department of Physics and Astronomy, Institute of Applied Physics, Research Institute of Advanced Materials (RIAM), Seoul National University, Seoul 151-747, Korea*

[4]*Department of Nanomaterials Science and Technology, University of Science and Technology, Daejeon, 305-333, Republic of Korea*

[5]*Department of Physics, Konkuk University, Seoul 143-701, Korea*

[6]*Department of Physics, The Catholic University of Korea, Bucheon 420-743, Korea*

[7]*Department of Physics and Astronomy, University of Kentucky, Lexington, KY 40506, USA*

[*]Correspondence and requests for materials should be addressed to Suyoun Lee (email: slee_eels@kist.re.kr)

S1. Examination of homogeneity of a Nb:STO film by SEM/EDX

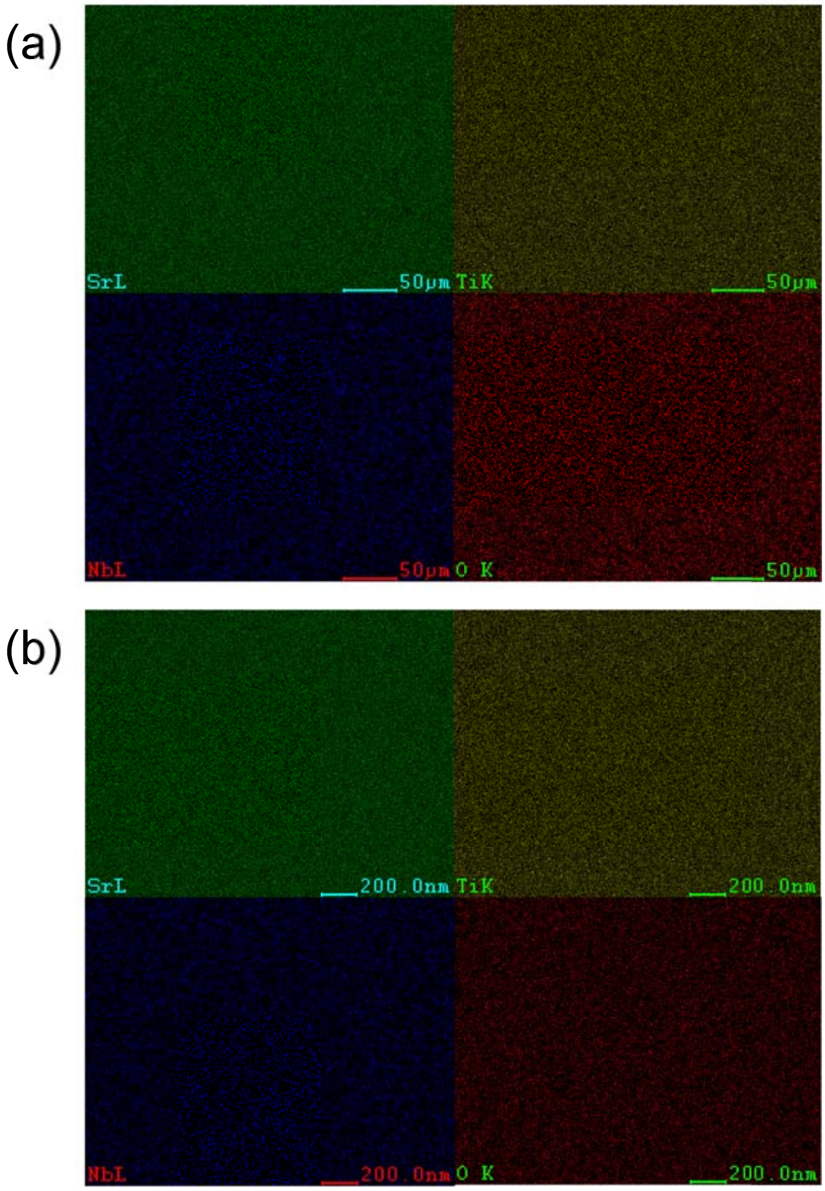

Figure S1. SEM/EDX images of a Nb:STO film at a low magnification (×600) (a) and a high magnification (×100,000) (b). It can be seen that all elements are uniformly distributed meaning that the observed LMR is not attributed to the inhomogeneity of the film.

S2. Reproducibility of the linear MR of other samples

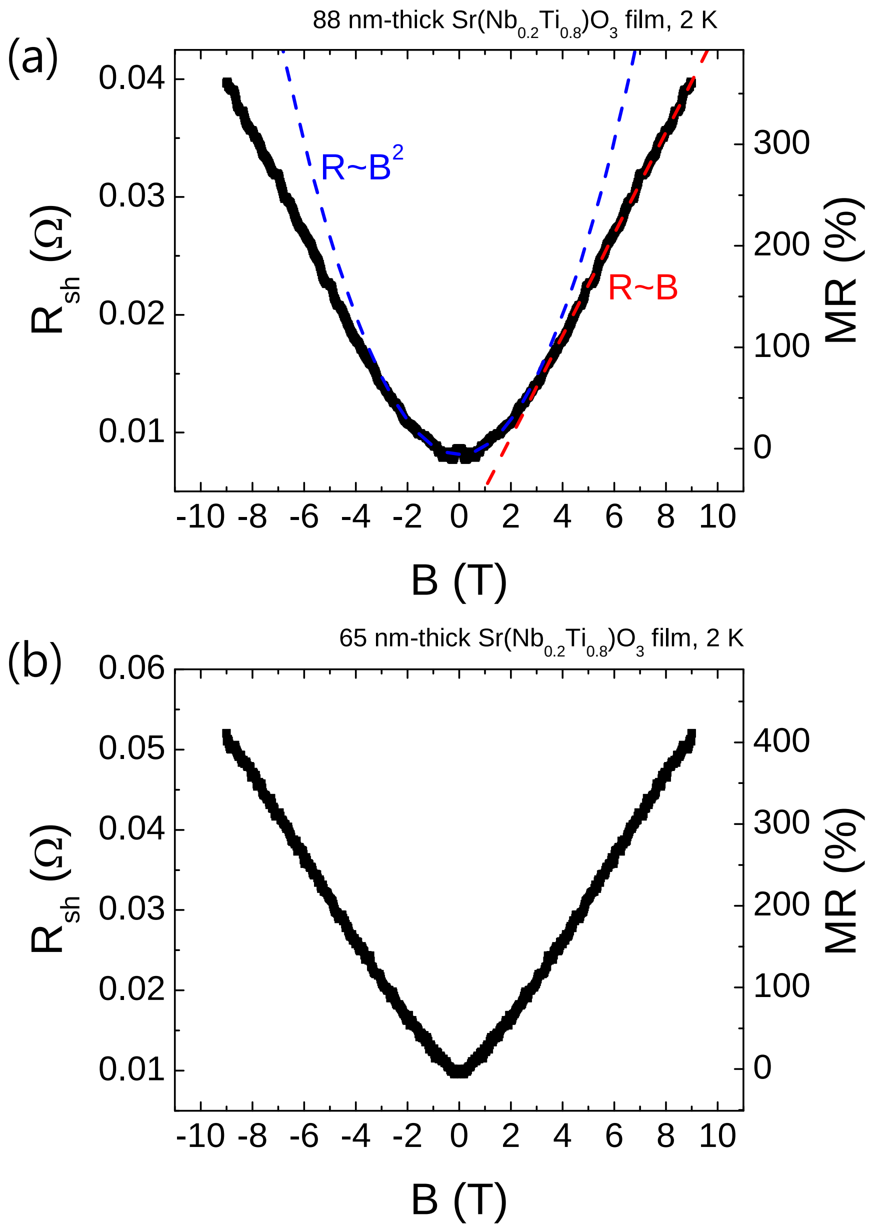

Figure S2. MR as a function of *B* of other $Sr(Nb_{0.2}Ti_{0.8})O_3$ films with different thickness ((a): 88 nm, (b): 65 nm). In (a), blue and red dashed lines are fitting curves with the quadratic *B*-field dependence at low field region (blue: -2~2 T) and a linear *B*-field dependence at high field region (red: 3~9 T), respectively.

S3. Magnetic property of a Nb:STO film

Figure S3(a) shows the temperature dependence of the magnetization (*M*) of a Nb:STO film as a function of temperature (*T*) with the external magnetic field (*H*) of 7 T being applied. ZFC represents the zero-field-cooled state, which is measured by cooling the specimen down to 2 K under zero magnetic field and heating the specimen under 7 T. And FC represents the field-cooled state, which is measured by cooling the specimen down to 2 K under 7 T and heating the specimen under the same field. Both of ZFC and FC curves show a drastic increase in *M* below 10 K indicating the occurrence of the magnetic ordering. Figure S3(b) shows *M* as a function of *H* at 5 K, where a small remnant magnetization is observed supporting the emergence of a magnetically ordered phase. These observations seem to be correlated with the resurgence of resurgence of $n(T)_{5T}$ below 10 K shown in Fig. 2(f) in the main text.

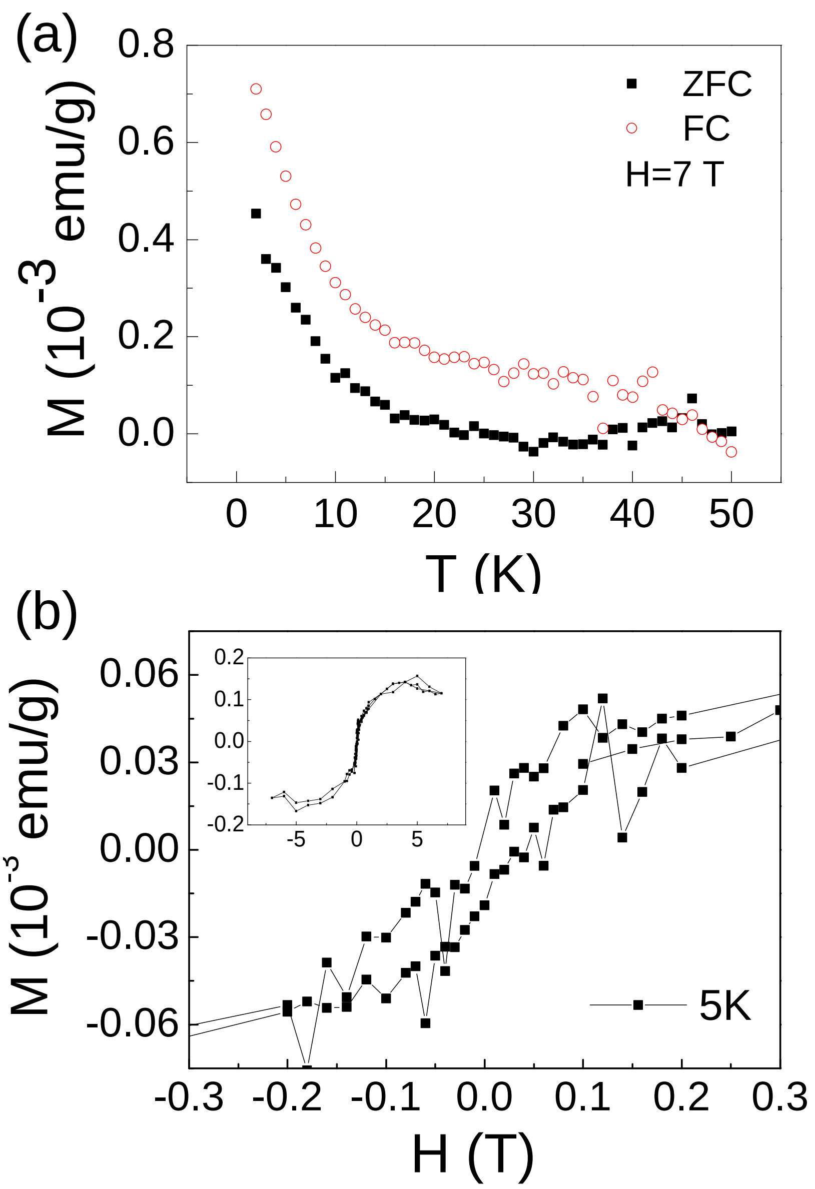

Figure S3. (a) Magnetization (*M*) of a Nb:STO thin film as a function of *T*. *M* was measured with the applied magnetic field (*H*) of 7 T. ZFC and FC represent zero-field-cooled *M* and field-cooled *M*, respectively. (b) *M* vs. *H* curve at 5 K. The inset shows the same curve in the full scale.

S4. A plot of the normalized MR vs. *B*

In Figure S4, $[\rho(B)/\rho(0)]*[k_B T/\mu_B B]$ for $B//\hat{n}$ has been plotted as a function of B with varying T using the data in Figure 2(b) in the manuscript, where μ_B is the Bohr magneton, respectively. According to the QLMR picture, this quantity is known to approach a constant value, which depends on the material, in the quantum limit and does not depend on T. It is clearly seen that the quantity converges to a constant value below 10 K at high B regardless of T and B.

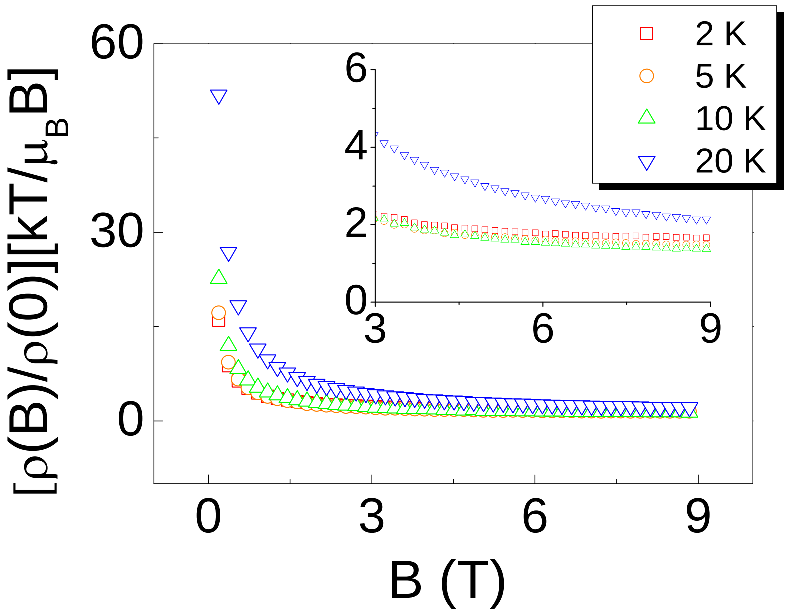

Figure S4. Normalized MR ($=[\rho(B)/\rho(0)]*[k_B T/\mu_B B]$) as a function of B at various temperatures. (Inset) A magnified view in the range of B=3~9 T.

S5. SdH oscillation in heaviliy-doped Nb:STO thin films

In Figure S5, we have plotted the second derivative of R_{xx} with respect to B as a function of $1/B$ to find a signature of the Shubnikov-de Haas (SdH) oscillation. (a) and (b) show the plot for a 65-nm thick film under B perpendicular to the plane and under B parallel to the plane but orthogonal to the current, respectively. In both of them, any distinguished periodic oscillation is not found indicating the absence of the SdH oscillation although the amplitude of the oscillation (or fluctuation) is found to increase at high magnetic field in the case of (b). Consequently, we couldn't distinguish the SdH oscillation in the 65-nm thick film. Meanwhile, we have presented the same kind of plot for a 10-unit cell thick film (Figure S5(c)), which shows a much higher MR (~1,800 % at 9 T, 1.8 K) and the much higher carrier mobility (~37,000 cm^2/Vs at 1.8 K). Although the curves are still noisy, several peak and dip structures are clearly distinguishable at 1.8 K. Among those, three peak and dip structures (indicated by blue arrows) are seen to be periodic in $1/B$ scale with their amplitude increasing with the strength of B, suggesting the possibility of the SdH oscillation as their origin. To confirm the SdH oscillation, we have investigated the same plot at 3 K and 5 K. As shown in the figure, the peak and dip structures at $1/B$=0.125 (T^{-1}) and 0.14 (T^{-1}) are reproduced at 3 K while a peak and dip structure is not reproduced at $1/B$=0.146 (T^{-1}). And, at 5 K, all the peak and dip structures have disappeared. In conclusion, we reasonably think them as the signature of the SdH oscillation although we don't have a decisive evidence for it because of the lack of the number of oscillations. About the reason for the ill-defined observation of the SdH oscillation despite such a high carrier mobility, we suspect the broadening of the Landau level which is thought to be comparable to the thermal energy around 1.8 K, making the Landau levels not clearly separated from each other.

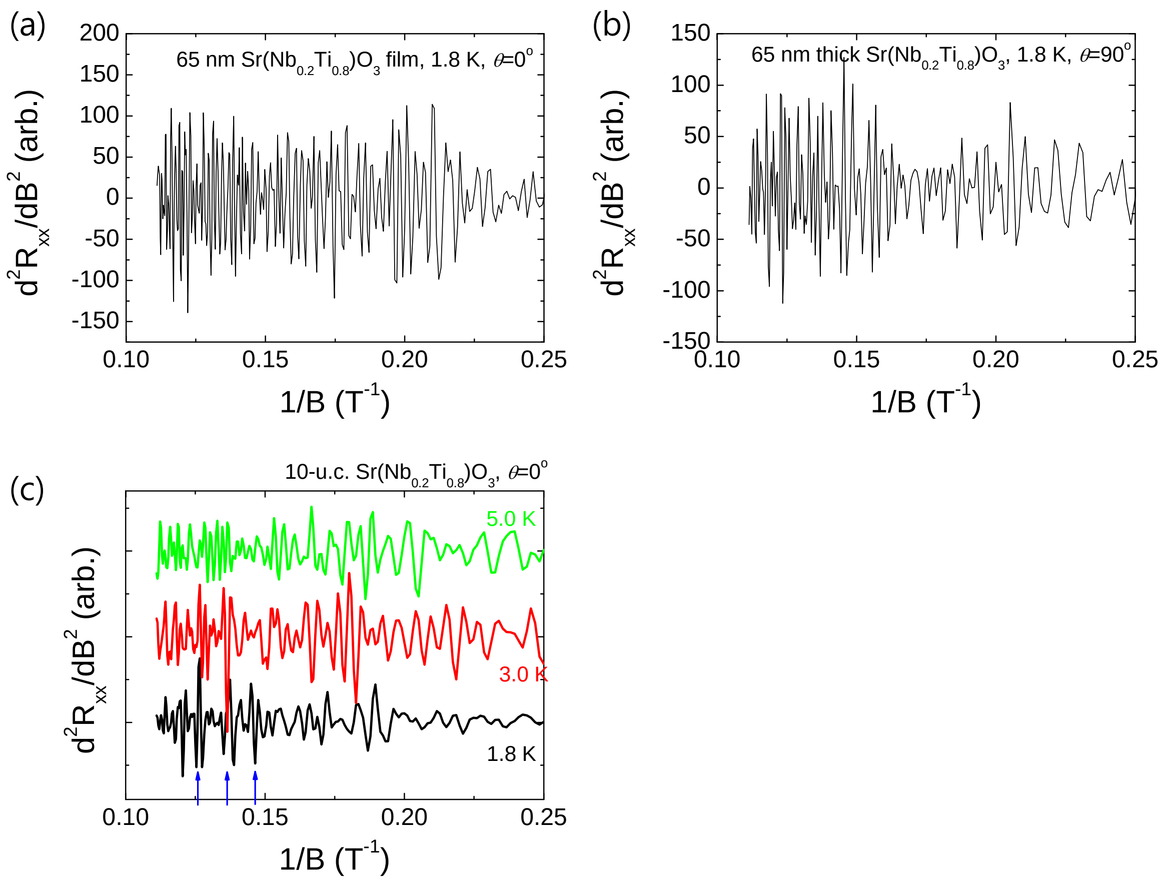

Figure S5. **Plot of d^2R_{xx}/dB^2 as a function of $1/B$.** (a), (b) For a 65-nm thick $Sr(Nb_{0.2}Ti_{0.8})O_3$ film under B perpendicular to the plane (a) and B parallel to the plane, but orthogonal to the current, (c) For a 10-unit cell (u.c.) thick $Sr(Nb_{0.2}Ti_{0.8})O_3$ film under B perpendicular to the plane at 1.8 K (black), 3 K (red), and 5 K (green). Blue arrows in (c) indicate oscillations with the amplitude relatively larger than the surroundings, indicating a possible signature of the Shubnikov-de Haas oscillation.
